# Dysbiosis-Associated Polyposis of the Colon—Cap Polyposis

**DOI:** 10.3389/fimmu.2018.00918

**Published:** 2018-05-07

**Authors:** Kazuki Okamoto, Tomohiro Watanabe, Yoriaki Komeda, Ayana Okamoto, Kosuke Minaga, Ken Kamata, Kentaro Yamao, Mamoru Takenaka, Satoru Hagiwara, Toshiharu Sakurai, Tomonori Tanaka, Hiroki Sakamoto, Kiyoshige Fujimoto, Naoshi Nishida, Masatoshi Kudo

**Affiliations:** ^1^Department of Gastroenterology and Hepatology, Kindai University Faculty of Medicine, Osaka-Sayama, Japan; ^2^Department of Pathology, Kindai University Faculty of Medicine, Osaka-Sayama, Japan; ^3^Department of Gastroenterology and Hepatology, Katsuragi Hospital, Kishiwada, Japan

**Keywords:** cap polyposis, intestinal microbiota, next-generation sequencing, inflammation, antibiotics

## Abstract

Cap polyposis is a rare gastrointestinal disease characterized by multiple inflammatory polyps located between the distal colon and the rectum. Despite the lack of clarity regarding its pathogenesis, mucosal prolapse, chronic inflammatory responses, and *Helicobacter pylori* infection are considered key contributors to the development of this disease entity. Although it is now generally accepted that dysbiosis of gut microbiota is associated with intestinal and extra-intestinal diseases, alterations of intestinal microbiota have been poorly defined in cap polyposis. Here, we report a patient with *H. pylori*-negative cap polyposis who was successfully treated with antibiotics and exhibited dramatic alterations in intestinal microbiota composition after antibiotic treatment. The patient was treated with oral administration of ampicillin and metronidazole and showed regression of cap polyposis 6 months after antibiotic treatment. Fecal microbiota analysis using the next-generation sequencing technology revealed a significant alteration in the intestinal microbiota composition following antibiotic treatment—a marked reduction of *Blautia, Dorea*, and *Sutterella* was observed concomitant with a marked increase in *Fusobacterium*. These data suggest that cap polyposis may originate from dysbiosis and that microbiome-targeted therapy may be useful in this disorder.

## Highlights

- Cap polyposis patient.
- Cap polyposis is considered as multiple inflammatory polyps.
- Regression of colonic polyposis after antibiotic treatment.
- Next-generation sequencing reveals dynamic changes in the intestinal microbiota composition following antibiotic treatment.
- Identification of pathogenic bacteria associated with cap polyposis.

## Introduction

Cap polyposis is a rare disease characterized by multiple inflammatory polyps that are covered by a cap of fibrinopurulent mucus and are located between the distal colon and the rectum (1–3). Patients usually present with abdominal pain, blood and/or mucus in diarrheal stool, and hypoproteinemia (1–3). Microscopically, the colonic polyps are characterized by a cap of fibrinopurulent exudates, distorted glands, fibromuscular obliteration of the lamina propria with inflammatory cell infiltration (1–3). Although the pathogenesis of cap polyposis remains unknown, the clinical and histopathological features of this disorder resemble those observed in patients with mucosal prolapse syndrome (MPS) (4). Therefore, mucosal prolapse secondary to impaired colonic motility has been considered a possible etiological contributor to cap polyposis (4). Several reports have shown regression of cap polyposis following the use of infliximab (2) or steroid (3), thereby demonstrating the possible involvement of chronic inflammatory responses in the development of cap polyposis. Reportedly, eradication of *Helicobacter pylori* is effective in the management of cap polyposis (5, 6). However, *H. pylori* were not detected in the colonic mucosa in any of these cases. Therefore, it is possible that unidentified intestinal bacteria, sensitive to *H. pylori-*eradication therapy, may contribute to the development of cap polyposis. Considering that eradication of *H. pylori* causes a significant alteration in the intestinal microbiota composition, these case reports suggest that dysbiosis-related immune responses may underlie the pathogenesis of cap polyposis. However, the intestinal microbiota composition has not been determined in this condition.

We report a patient with *H. pylori*-negative cap polyposis who was successfully treated using antibiotics. Fecal microbiota analysis using the next-generation sequencing (NGS) technology revealed a significant alteration in the intestinal microbiota composition pre- and post-antibiotic treatment. The results of our study strongly support the contributory role of dysbiosis in the pathogenesis of cap polyposis.

## Case Report

**Figure 1:**
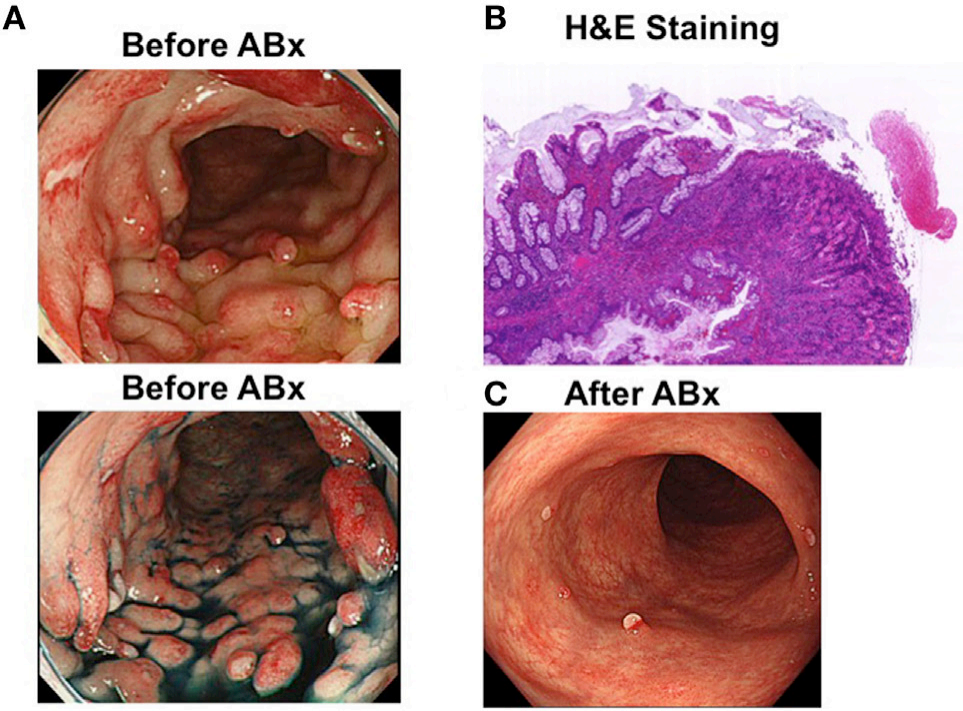
Endoscopic and pathological findings in a patient with cap polyposis. **(A)** Endoscopic images in a patient with cap polyposis before antibiotic treatment (before ABx). Multiple sessile polyps are observed in the descending colon prior to the initiation of antibiotic treatment. Top and bottom panels show white light endoscopy and chromoendoscopy images, respectively. The polyps appear reddish in color and are covered by white mucus. **(B)** Microscopic pictures of a patient with cap polyposis. Endoscopic mucosal resection was performed to obtain a histopathological diagnosis. Low magnification revealed inflammatory polyps covered with granulation tissue and fibrinopurulent exudate. Distorted glands were also seen in lamina propria. Hematoxylin and eosin (H&E) staining. Magnification ×40.**(C)** Endoscopic images obtained from a patient with cap polyposis treated with antibiotics (after ABx). Most of the inflammatory polyps are observed to have disappeared 6 months post-antibiotic treatment.

An asymptomatic 45-year-old man without a relevant past or family history of gastrointestinal disease underwent a colonoscopic and esophagogastroduodenoscopic examination for the evaluation of a positive fecal occult blood test. Colonoscopic examination revealed multiple sessile polyps in the descending colon, which showed a reddish surface covered by white mucus (Figure 1A).Esophagogastroduodenoscopic examination revealed multiple fundic gland polyps. Serum anti-*H. pylori* antibody titers were below the detection limit, and serum total protein and albumin levels were within the reference range, as was the complete blood cell count. Endoscopic mucosal resection was performed to determine a histopathological diagnosis of the colonic polyps. The resected specimen showed mucus-containing distorted glands and significant inflammatory cell infiltration with fibrosis in the lamina propria (Figure 1B) and their surface was covered by inflammatory granulation tissue and fibrinopurulent exudate. These endoscopic and histopathological findings were consistent with those typically observed in patients with cap polyposis (1–3). Thus, this patient was diagnosed with cap polyposis without *H. pylori* infection.

*Helicobacter pylori* infection has been considered a possible etiological contributor to the development of cap polyposis because eradication of this organism is observed to cause regression of colonic polyps in some patients (5, 6). However, notably, *H. pylori* have not been detected in the colonic mucosa in any patient diagnosed with cap polyposis. Thus, gut bacteria sensitive to the antibiotic component of *H. pylori-*eradication therapy are likely to play a pathogenic role in patients with cap polyposis. Based on this hypothesis, this patient was treated with oral administration of ampicillin (1,500 mg/day) and metronidazole (500 mg/day) for 1 week, and regression of cap polyposis was observed 6 months post-antibiotic treatment (Figure 1C). This clinical course strongly suggests that antibiotic-induced eradication of pathogenic gut bacteria responsible for the development of inflammatory polyps can cause regression of cap polyposis.

## Fecal Microbiota Analysis

Stool samples pre- and post-antibiotic treatment were subjected to fecal microbiota analysis, which was performed as previously described (7, 8) to assess any alterations in the intestinal microbiota composition. Ethical approval for this study was granted by a Review Board of the Kindai University Faculty of Medicine. DNA samples extracted from the stool were subjected to polymerase chain reaction for the amplification of the 16S ribosomal RNA (16S rRNA) V3 and V4 regions. Primer sequences are available in our previous report (8). We performed 16S rRNA sequencing using the MiSeq system (Illumina) (7, 8). Trimmomatic, Cutadapt, and Fastq-join programs were used for sequence data processing, and operational taxonomic units (OTUs) were defined using the QIIME program (7, 8). The defined OTUs were subjected to population analysis to identify the bacterial phylum, class, order, family, and genus.

**Figure 2:**
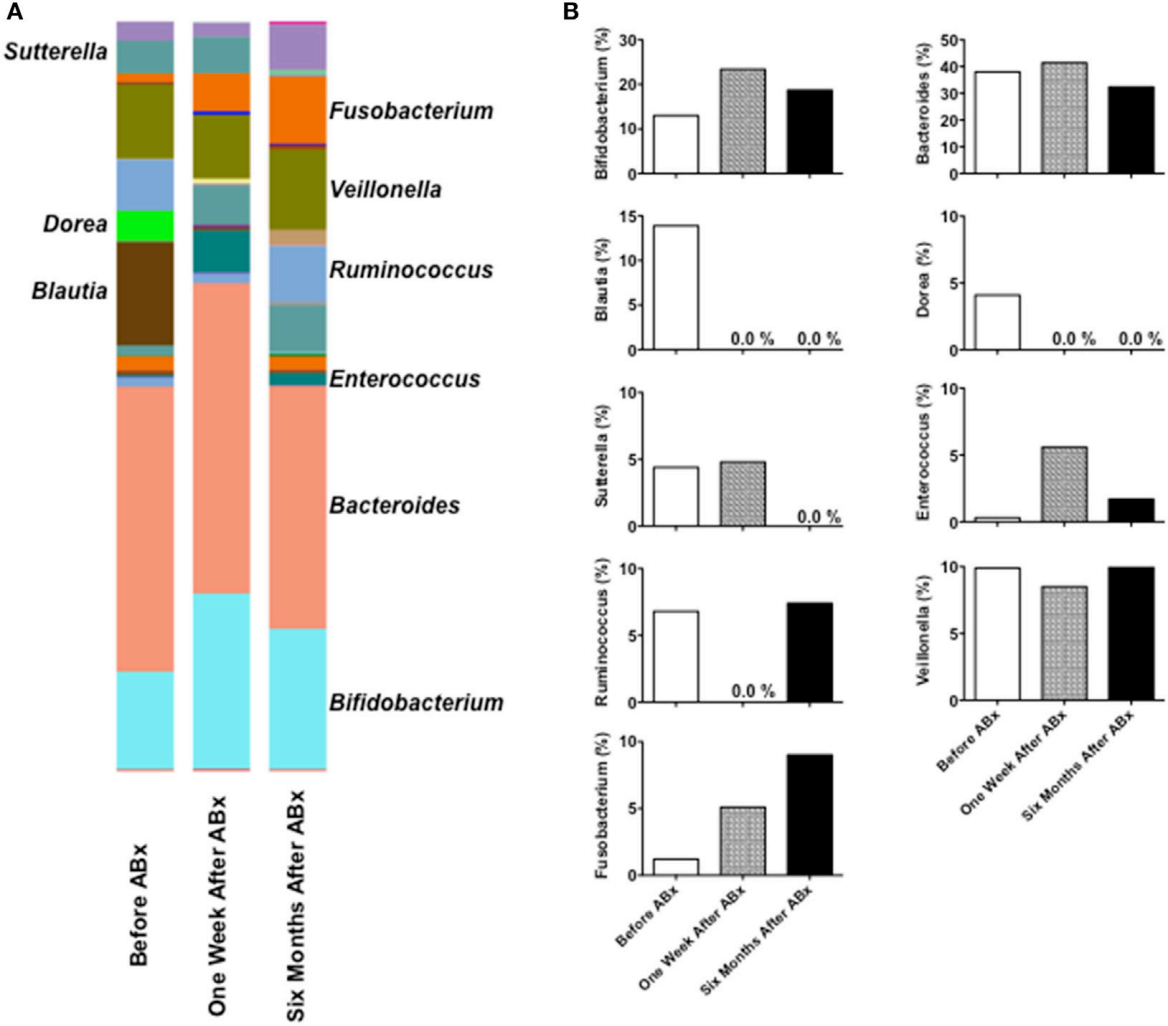
Fecal microbiota analysis in a patient with cap polyposis. **(A)** Stool samples were obtained from a patient with cap polyposis prior to, 1 week and 6 months post-antibiotic treatment. DNA samples extracted from the stool specimens were subjected to polymerase chain reaction for the amplification of the 16S ribosomal RNA (16S rRNA) V3 and V4 regions. We performed 16S rRNA sequencing using the MiSeq system. The relative abundance of different bacterial taxa at the genus level in each sample has been shown. **(B)** Comparative analysis of the taxonomic composition of the fecal microbial community at the genus level. Relative abundance of the genera has been shown as a percentage.

We detected 1,077 OTUs from 3 stool samples obtained pre- and post-antibiotic treatment. No major taxonomic alterations in the microbial communities were observed at the level of the phylum, order, or family (data not shown). Significant changes in the composition of fecal microbiota were noted at the genus level pre- and post-antibiotic treatment (Figure 2A). *Blautia* and *Dorea*, which showed a high relative abundance in the feces pre-antibiotic treatment, disappeared 1 week and 6 months post-antibiotic treatment (Figure 2B), and *Sutterella* disappeared 6 months post-antibiotic treatment. By contrast, the relative abundance of *Fusobacterium* was observed to have increased post-antibiotic treatment. The relative abundance of *Bifidobacterium, Bacteroides*, or *Veillonella* remained largely unchanged pre- and post-antibiotic treatment. This microbiota analysis suggests that regression of cap polyposis following antibiotic treatment is accompanied by a marked decrease in *Blautia, Dorea*, and *Sutterella* and a marked increase in *Fusobacterium*, indicating that cap polyposis might have originated from dysbiosis in this patient.

## Discussion

Recent progress in NGS technology has highlighted the role of an altered intestinal microbiome (dysbiosis) in human diseases (9). Inflammatory bowel disease (IBD) is a prototypical dysbiosis-related disorder mediated by abnormal immune responses to altered intestinal microbiota (9). In this study, we report a patient with cap polyposis in whom antibiotic treatment resulted in regression of multiple inflammatory polyps. Interestingly, regression of cap polyposis was associated with significant alterations in the composition of the intestinal microbiota. Thus, we propose that cap polyposis might be a dysbiosis-related intestinal disorder. It should be noted, however, that we cannot exclude two other possibilities in the regression of cap polyposis. First, spontaneous regression might have occurred in antibiotic treatment-independent manner as previously reported (3). Second, anti-inflammatory responses leading to the regression of cap polyposis might be induced by antibiotic treatment.

Mucosal prolapse syndrome and cap polyposis share endoscopic findings in that reddish elevated mucus-covered lesions are common to both conditions (4). Histopathologically, both disorders are characterized by findings of superficial erosions covered by inflammatory granulation tissue, distorted and elongated glands, and fibromuscular obliteration of the lamina propria—all these being typical findings observed in our patient. These clinical and histopathological similarities lead us to the conclusion that MPS and cap polyposis share common pathogenetic mechanisms—mucosal prolapse secondary to impaired colonic motility noted in MPS has been considered a possible etiological factor in cap polyposis. However, MPS and cap polyposis differ in terms of treatment because suppression of a chronic inflammatory response using steroid or infliximab is often effective only in the latter (2, 3). Moreover, antibiotic-induced eradication of *H. pylori* or as yet unidentified bacteria can lead to regression of cap polyposis (5, 6). These reports strongly indicate the role of chronic immune reactions toward intestinal microflora in the pathogenesis of cap polyposis. Therefore, cap polyposis might be caused by impaired host-bacterial mutualism. We have demonstrated that antibiotic treatment leads to regression of cap polyposis through significant alterations in fecal microbiota composition. Our results strongly support the idea that impaired host-bacterial mutualism caused by dysbiosis underlies the pathogenesis of cap polyposis. Further studies are warranted to assess the intestinal microbiota composition in a larger number of samples with cap polyposis to validate our results.

Significant changes in fecal microbiota composition were observed at the genus levels pre- and post-antibiotic treatment. The relative abundance of *Blautia, Dorea*, and *Sutterella* was markedly decreased 6 months post-antibiotic treatment compared to the pre-antibiotic treatment finding, whereas the relative abundance of *Fusobacterium* was markedly increased post-antibiotic treatment. Thus, disappearance of *Blautia, Dorea*, and *Sutterella* and colonization of *Fusobacterium* were observed to be associated with regression of cap polyposis. Therefore, *Blautia, Dorea*, and *Sutterella*, and *Fusobacterium* might be considered pathogenic and beneficial bacteria, respectively, for cap polyposis. Consistent with these results, Nishino et al. have shown that mucosal microbiota composition in those with ulcerative colitis is characterized by a greater abundance of *Blautia* (10). An increased abundance of *Dorea* has been detected in the normal colonic mucosa in patients with colorectal adenomas (11). However, an increased percentage of *Blautia* and *Dorea* observed in the gut mucosal microbiota composition is reportedly associated with remission after surgery in IBD patients (12, 13). Mukhopadhya et al. have reported that *Sutterella* is unlikely to play a role in the pathogenesis of IBD (14). Moreover, *Fusobacterium*, which demonstrated higher relative abundance post-antibiotic treatment in this patient, has been shown to promote colorectal tumor growth (15). Although the discrepancy between our data and previous reports remains unexplained, it could be attributed to a likely difference between fecal and mucosal microbiota composition. Identification of pathogenic or beneficial bacteria for cap polyposis requires future studies that assess microbiota composition in a larger number of patients with cap polyposis. Fecal microbiota analyses in a large number of patients with cap polyposis are necessary to confirm the involvement of dysbiosis and to determine the sensitivity to antibiotic treatment in this disorder.

## Concluding Remarks

To our knowledge, this is the first report describing intestinal microbiota analysis in a patient with cap polyposis. Our results strongly suggest that cap polyposis may originate from dysbiosis and that microbiome-targeted therapy may be useful in this disorder.

## Ethics Statement

This study was carried out in accordance with the recommendations of a Review Board of the Kindai University Faculty of Medicine with written informed consent from the patient. The patient gave written informed consent in accordance with the Declaration of Helsinki. The protocol was approved by a Review Board of the Kindai University Faculty of Medicine.

## Author Contributions

KO, TW, YK, AO, HS, and KF took care of the patient. KO, TW, KM, KK, KY, MT, SH, and TS wrote the manuscript. TT performed pathological examinations. KO, TW, and NN performed experiments. MK supervised the research. All the coauthors checked the final version of the manuscript before the submission.

## Conflict of Interest Statement

The authors declare that the research was conducted in the absence of any commercial or financial relationships that could be construed as a potential conflict of interest. The reviewer MM and handling Editor declared their shared affiliation.

## Abbreviations

IBD, inflammatory bowel disease; MPS, mucosal prolapse syndrome; NGS, next-generation sequencing; OTU, operational taxonomic unit; rRNA, ribosomal RNA.
